# Triple-crises-induced food insecurity: systematic understanding and resilience building approaches in Africa

**DOI:** 10.1016/j.soh.2023.100044

**Published:** 2023-10-29

**Authors:** Ernest Tambo, Chen-Sheng Zhang, Gildas B. Tazemda, Bertin Fankep, Ngo T. Tappa, Cremona F Bette Bkamko, Laura M. Tsague, Daniella Tchemembe, Elodie F. Ngazoue, Kennedy K. Korie, Marie Paule N. Djobet, Oluwasogo A. Olalubi, Omer N. Njajou

**Affiliations:** a Higher Institute of Health Sciences, Faculty of Medicine, Universite des Montagnes, Cameroon; b Center for Leadership in Global Health Equity, University of Global Health Equity, Kigali, Rwanda; c Africa Disease Intelligence, Preparedness and Response (ADIPaR), Yaoundé, Cameroon; d National Institute of Parasitic Disease, Chinese Centre for Disease Control and Prevention (Chinese Center for Tropical Diseases Research), Shanghai, China; e Institut Universitaire et Stratégique de l'Estuaire, Institut des Sciences Appliquées à la Santé (IUEs/INSAM/ISSAS), Cameroon; f Department of Public Health, Faculty of Medicine, University of Douala, Cameroon; g Labotaroire d’Analyse et d’Expertise Biomédicales (LaboreB), Cameroon; h Department of Public Health, Faculty of Basic Medical Sciences, Kwara State university (KWASU), Malete, Nigeria; i DAI Tackling Deadly Diseases in Africa (TDDA) Programme, Cameroon

**Keywords:** Food insecurity, One Health, Preparedness, Policies, Actions, Resilience, Supply chain, Consumption, Africa

## Abstract

The triple crises of the COVID-19 pandemic, conflict and climate change have severely impacted food systems, leading to socio-economic consequences and undermining food and nutrition security across Africa. To address the malnutrition and poverty affecting approximately 700 million people in Africa, there is potential for the One Health approach implementation and operationalization to bring together multidisciplinary solutions for tackling food insecurity and ensuring food safety net. However, there is limited documentation on the potential of the One Health approach system thinking implementation to guide responses to triple crises-induced food insecurity. Therefore, this article aims to systematically understand the triple crises-induced food insecurity, connect existing solutions, and explore the role of the One Health approach in strengthening food and agriculture systems in Africa.

Our finding showed the impact of triple crises exacerbating food system vulnerability in Africa and worldwide. Mitigating and resilient actions are urgently needed in tackling the emerging and persisting challenges, and infectious diseases menace and burden across Africa. We present a conceptual model illustrating the complex nature of triple crises-induced food insecurity, vulnerability areas within the food system, and actionable strategies for building community food resilience. Additionally, recommendations are provided to create an enabling environment that supports One Health approach implementation and addresses food insecurity challenges through innovative partnerships, local-led initiatives, and enhanced governance and artificial intelligence technology capacities in achieving sustainable and inclusive growth to reduce socio-economic inequalities.

Stepping up integrated, actionable, and sustainable food systems programs and innovative long-lasting solutions requires investing in promoting new partnership and research collaboration in building conflict resolution and peace towards strengthening and reshaping local and global food security related climate change adaptations actions for most vulnerable communities’ benefits. These are ingredients in fastening preparedness, prevention and control of infectious diseases prevention and control, reducing food supply chains disruption towards accelerating equitable benefits of Universal Health Coverage and Sustainable Development Goals, 2030 across Africa.

## 1 Introduction

Food systems have been adversely impacted by the climate-induced shocks, conflicts and coronavirus disease 2019 (COVID-19) pandemic crises. These triple crises have resulted in an unprecedented socio-economic effect, and eroded the resilience of large segments of the poor and marginalized population, and threatened to wipe out the gains made towards achieving food and nutrition security in Africa and elsewhere [1,2]. COVID-19 pandemic lockdown and containment further aggravated increasing competition for water, pasture and land, volatile security situations all of which are essential assets in promoting food systems. These persisting situations continue to jeopardize Africa hard-won food security and health gains over recent years.

Appropriate and holistic adaptive response measures are urgently needed for building a robust and resilient food system supplying sufficient, affordable food at all times in the world. The increasing recurrence of intense heatwaves with temperature over 40 °C, increasing droughts, forest fires and emerging/re-emerging pests are a constant reminder that our food system is under threat and must become more sustainable and resilient response actions [3]. Africa is experiencing a devastating recession after nearly three decades of relatively consistent development gains. The socio-economic shocks sparked by the COVID-19 pandemic and conflict crises brought Africa's GDP to fall by over 3% in 2020 and plunged more than 40 million Africans into poverty [2,3]. The unprecedented levels of armed violence, civil unrest and insecurity across West, Central and Sahel and East Africa drive millions displaced populations to the brink of starvation and hunger. As affected smallholder dairy farmers sell off livestock due to crises, the local supply of milk and cheese is declining; reduced consumption of these important foods worsening malnutrition, stunting in children, and lower productivity levels among the affected communities in the long term [[4], [5], [6]]. If not addressed promptly, the food insecurity might hinder the three key areas supporting Africa's development plans in a post-COVID-19 recovery era: promoting sustainable and inclusive economic growth; building resilient and secure societies; equity and establishing lasting peace and stability [7]. Building more resilient and sustainable food and nutrition systems, as well as implementing climate adaptation measures, is of primary importance to tackling the persisting malnutrition and poverty vicious cycle that affects nearly 700 million people in Africa [4]. Increasing the risk of early pregnancy, early forced marriage, school dropout, risk of sexual and gender violence are deepening lack of access to livelihoods and poverty, and increasing humanitarian needs [3,6].

This unprecedented level of threats to Africa's food system, as a wake-up call, demonstrates how health, ecosystems, and food system are all interlinked, proposing a complex problem that is urgently needed to be solved by One Health [8]. The One Health approach, endorsed by international organizations such as Food and Agriculture Organization of the United Nations (FAO), World Organization for Animal Health (WOAH), World Health Organization (WHO), and United Nations Environment Programme (UNEP), brings together multidisciplinary teams to tackle food insecurity [9]. In Africa, the implication of One Health approach are evident in agricultural collaboration efforts such as building sustainable food system with innovative partnership and resilient transformation: the Africa Union Agricultural path and Maputo declaration, which highlight the potential for building sustainable food systems with innovative partnerships [6,10]. Moreover, One Health approach plays a crucial role in ensuring food safety, particularly in addressing zoonoses-related food safety crises. It enables the understanding of virus transmission dynamics, identifies gaps in biosecurity, and develops mapping and predictive models for pathogens evolution and diseases threat spread. These insights inform evidence and contextual-risk mitigation, communication and public engagement, equity and resilience building strategies against foodborne diseases outbreaks and other emerging food systems crises [11,12].

Though the importance of One Health approach in food safety and food security is well recognized, little is documented on the potential of One Health approach to guide the response to the triple-crises-induced food insecurity in Africa. Therefore, this study aims to (i) systematically understand the triple-crises-induced food insecurity and hunger, (ii) connect the existing solutions for curbing the vicious cycle of food insecurity under triple crises, and (iii) explore the operationalization of One Health approach to strengthen the food system resilience against the triple crises across Africa. Through a literature review guided by One Health thinking and supported by Theory of Change and Vulnerability/Resilience theory (Annex 1.), this study seeks to provide insights into addressing the challenges, and amplifying evidence-based and equitable food security and resilience responses/innovations amidst the challenges posed by triple crises in Africa.

## 2 Results

### 2.1 Systematic understanding of the triple crises-induced food insecurity in Africa

**Fig. 1:**
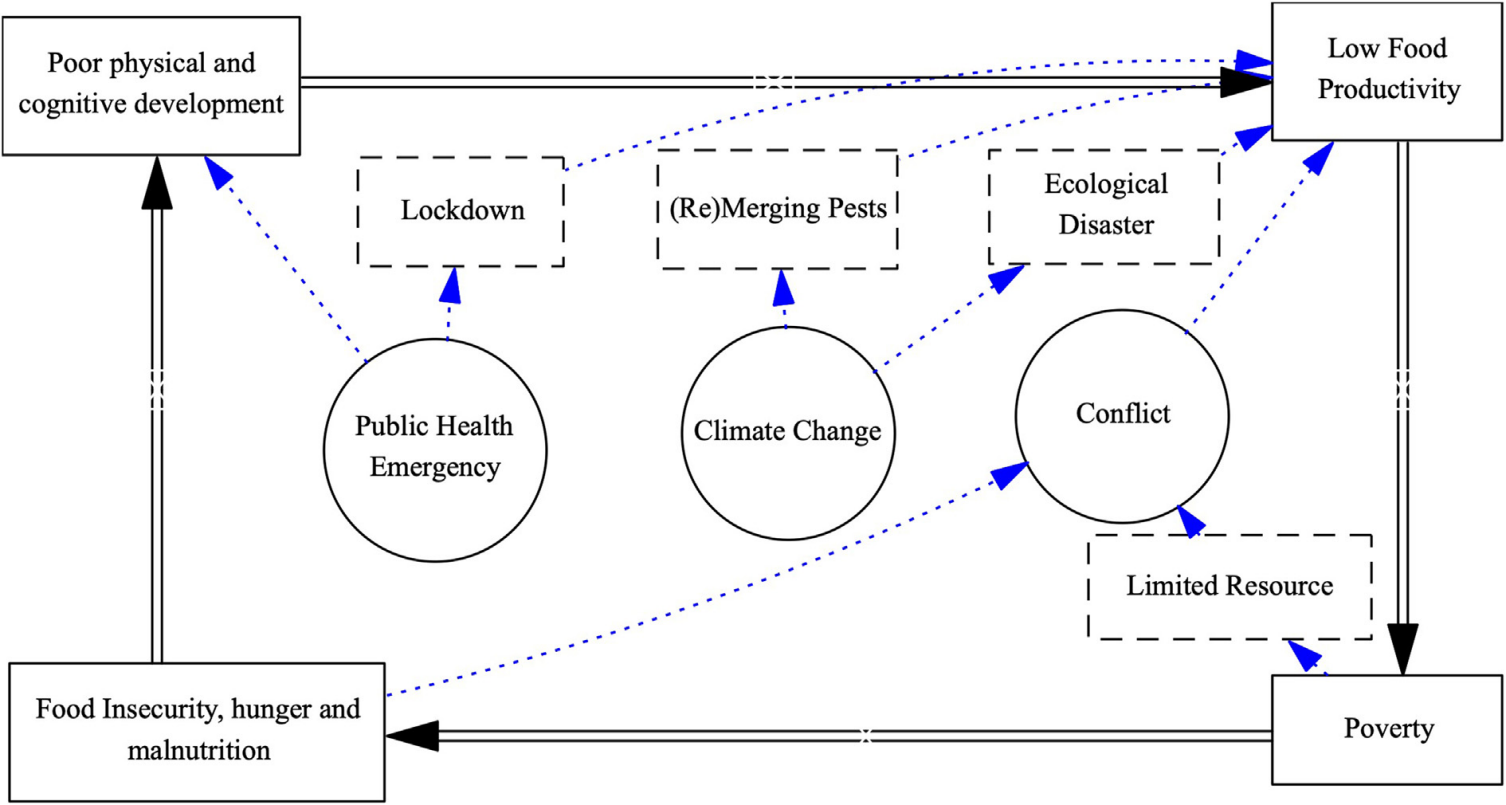
**Foodinsecuritylinkedtriplecriseseffects,** Modified by the authors from *FAO (2008). An Introduction to the Basic Concepts of Food Security. EC - FAO Food Security Programme.*https://www.fao.org/3/al936e/al936e.pdf.

The complex nature of triple-crises-induced food insecurity is summarized with evidence from the literature into the conceptual model in Fig. 1. This systemic conceptual model offers a practical and user-friendly tool for individual and governmental decision-makers to reach consensus on the long-term impact of the triple-crisis-induced food insecurity, and the necessity to interrupt the vicious cycle of food insecurity. It provides a better understanding of nexus thinking regarding linkages, tradeoffs, and synergies to mitigate the climate change, conflict, and COVID-19 impacts on food security, as well as the importance of building a resilient food system to support and better solutions for coping to safeguard ecosystems and food systems services.

This model illustrates that the crises can speed up the vicious cycle of food insecurity [13], by: (i) negative impacts of crises on the food system, which leads to the fewer meals and nutrition supplies; (ii) poor nutritional intake among workers would hinder the production, manufacture, and distribution within food system; (iii) poverty, resulting both from the negative impacts of crises and from a lacking nutrition supply, can exacerbate these negative impacts; and (iv) all of which can further lead to conflicts and social instability.

This visual representation reveals the interruption of the food system, especially the food production chain, is the key issue to tackle during crises. Past literature, as shown in Table 1, demonstrates that food productivity in West African countries was heavily affected by climate-change-linked disasters, conflict, COVID-19 cross-border lockdown measures, environmental degradation, agricultural risks, inflation of food prices, and gaps in resources related to poverty and public health emergencies. These effects are linked to food and water insecurity which exacerbate population vulnerabilities. Although various prospective solutions have been proposed, there has been a lack of systematic planning to halt the vicious cycle of food insecurity.

**Table 1:** Emerging food insecurity and hunger related global complex emergencies crises.

Global emergencies crises	Reported food insecurity effects	Prospective solutions and opportunities
**Climate change and greenhouse gases emission**	-Droughts and water scarcity -Increasing heatwaves, extreme heat and temperature -Increasing greenhouse gas emissions -Rising food commodities prices -Impacts on livelihoods -Failing to deliver on climate change Paris declaration and Sustainable Development Goals commitments by 2030 [2,3,8]	-Increasing investment in climate smart agriculture and food systems innovations -Promoting climate adaptation and clean energy solutions -Accelerating climate long-term food security policies and solutions -Boosting public-private partnership and strengthening research collaboration -Food data and knowledge sharing -Evidence-based, sustainable and transformation of food systems
**COVID-19 pandemic**	-Food chain supplies and distribution disruption -Restriction and closure of markets -Low food production and shortage o goods -Increasing food loss and waste -Increasing hunger and job loss/labor shortages -Food prices increase Markets inaccessibility [1,3,6].	-Develop and implement local food production systems and food security strategies -COVID-19 food and agriculture recovery programs -Scaling up innovative-based approach to Agric-technologies ecosystem ideas -Promoting healthier and sustainable food choices and food chain resilience -Evidence-based food resilience advocacy
**Conflict, political unrest, and war**	-Forced displacement abandon and of livelihoods and deaths -Displacement and destruction of agriculture assets -Food related humanitarian crisis -Rights and gender abuses Importation of food stuffs -Famine and malnutrition -Increasing food demand and price volatility -Reduction food supply [[3], [4], [5],^8^,^9^]	-Food aid and technical assistance in Agric-technologies -Long term agricultural productivity growth and local market development -Reduction of conflict in fragile communities, while promoting resilience and equity community of practice. -New investment and financing partnership in key agriculture and rural development areas for shared prosperity. - Increasing One Health workforce incentives, financial digital payment, and gender-based programs

The complex food crisis has been shown to trigger more widespread poor physical and cognitive development, low productivity, poverty-linked suffering and hunger, undermined agricultural and food systems, small farmer livelihoods, supply chain reduction and local food price inflation, all of which negatively affect food and nutrition security. All the socio-economic disasters can be traced back to the interruption of the food system, especially the food production and delivery chain.

**Fig. 2:**
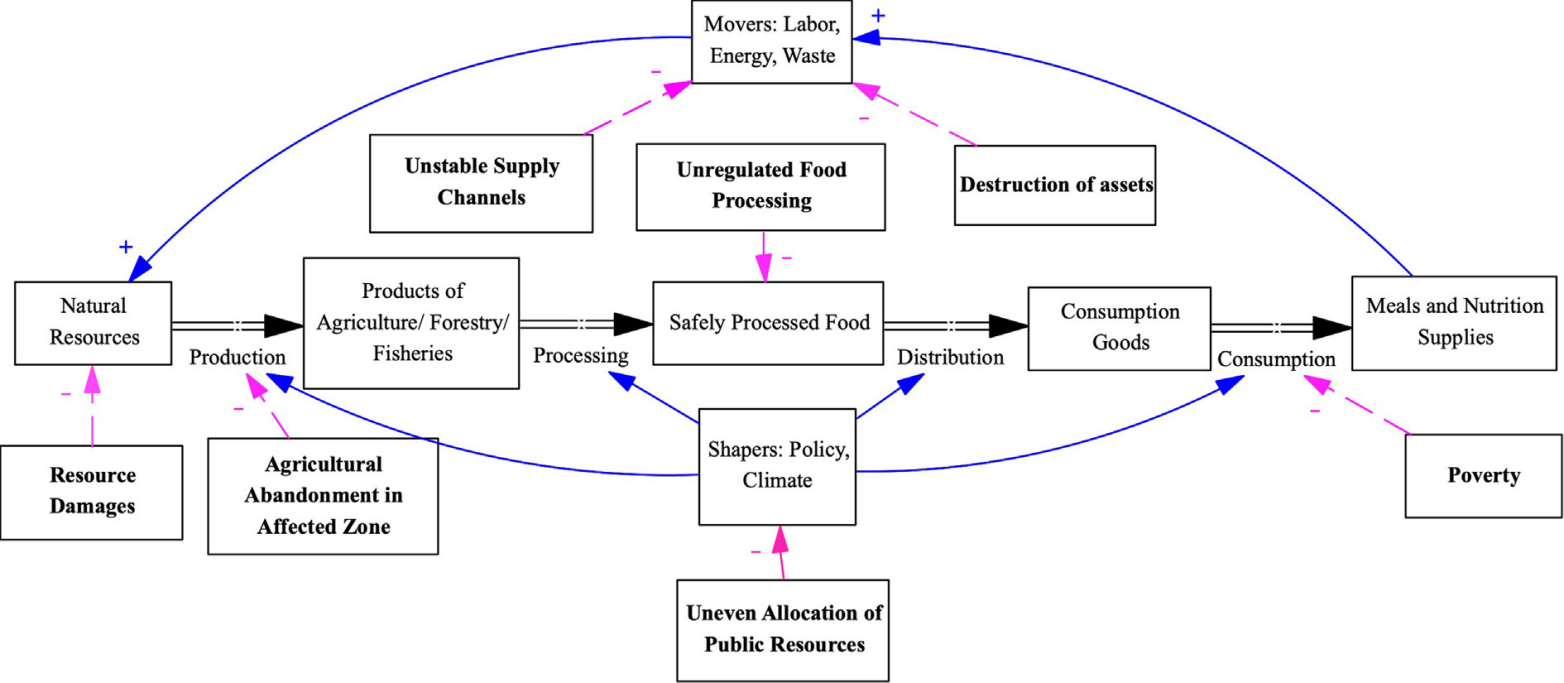
**The “Pathways of Impacts” of thetriple-crises-inducedfoodinsecurity,** The seven dotted lines illustrate the identified “pathways of impacts”; the structure of the food system (connected by solid directional lines) is modified by the authors from *Combs* et al. *1996. “Sustainable Food System Approaches to Improving Nutrition and Health”*, with Reference to *FAO. 2014. “Developing Sustainable Food Value Chain. Guiding Principles”.*

Thus, the next step is building from the consensus that to tackle the triple-crises-induced food insecurity, is to protect the food system under crises. From the literature review (Annex 1), the pathways of how crises impacted food systems have been identified with seven common “impact points” on the general food system model. Those “impact points” are the end of each “pathway of impact”, graphically presented in the Logic Model of the triple-crises-induced food insecurity (Fig. 2).

Scaling up responses and humanitarian investment commitment efforts are imperative through malnutrition screening and nutrition supplementation, voucher assistance, inclusive quality school meals and education programs, seed provision to farmers, government subsidizing fertilizers, and livelihood and protection support activities. To increase the readability of the Logic Model, the pathways of impact are shortened to the seven direct negative impact points shown in Fig. 2.

### 2.2 Analysis of food insecurity under triple crises in African region

Under the triple crises in concern, the endpoints of the seven “pathways of negative impacts” have been identified as the most vulnerable parts of the food system. Hence, the seven vulnerability areas revealed by how the seven “pathways of negative impacts” act on the food system. Given the interactions among the seven impact points, which share components with vulnerable areas of food system, we present each area of vulnerability once to avoid redundancy as the lack of economic or physical access to food sufficiency need daily, increasing hunger and malnutrition, extending to economic, social, and environmental and health effects undermine previous gains and post-COVID-19 recovery. The multifaceted web related policy, production to consumption equitable access and uptake challenges and consequences, call for a concerted response and resilience from governments, the private sector, stakeholders and communities.

1) *Unevenallocation ofpublicresources:* The “uneven allocation of public resources” could speed up the vicious cycle of food insecurity, if the shortage within the food supply chain is neglected. Since further impacts along the “pathways of negative impact” can happen with a time lag, “uneven allocation of public resources” often happens when the emergency response only focusing on the most visible impacts of the crises and neglecting the holistic and systematic changes [[14], [15], [16], [17], [18], [19]]. Unfortunately, this seemingly unpreventable temporary negligence in public governance can lead to all other areas of vulnerability (the other six pathways showing in Fig. 2), and cause a lasting effect on the food system(s) [[20], [21], [22], [23], [24]]. Thus, we identified evidence-informed policies and governance as the key area of vulnerability to food security under crises, requiring systematic approaches such as One Health to identify vulnerability in the food systems in time.
2) *Unstablesupplychannels*: “Unstable supply channel” hinders the “flow” of the food system. That is the transportation of raw materials is limited during crises, then ceasing the production, processing and consumption within the food system(s), and limiting access to meals and nutrition supplies [[25], [26], [27]]. Especially in the African region, as the majority of the population rely on imported food supplements, the disturbance in supply channels has left communities in low nutrition status and speeding up the vicious cycle of food insecurity [[28], [29], [30], [31], [32], [33], [34], [35], [36], [37], [38], [39]]. Thus, this highlights the vulnerability of food-related transportation within the food system under triple crises, underscoring the needed to be strengthened and guarded.
3) *Destruction ofassets*: “Destruction of assets” lowers the efficiency of turning raw materials into meals and other nutrition supplies, and might cause food insecurity with lower population nutrition levels [21,31,[40], [41], [42], [43], [44], [45], [46]]. Under crises, the workforce capacity and emerging technologies/tools can be harmed and decrease productivity [24,27,30,[47], [48], [49]]. If the key food production firms and agro-industries in the communities are compromised, the local population will have to seek external and unstable food sources [[50], [51], [52], [53]]. Thus, local food production entities, despite size and ownership, represent a vulnerable area of the food system(s), that require extra attention and supports (e.g. supporting fiscal policy, tax exemption, and coordinated investment efforts) during crises and recovery.
4) *Resourcedamages*: “Resource damages” challenge the material foundation of the food system in nature. Various natural resources are under threats of triple crises, ranging from clean water to farming animals. Conflicts, climate change, and COVID-19 pandemic, can all cause irreversible damage to the key resources of the food system(s) as mentioned above [26,34,37,[54], [55], [56], [57], [58]]. If those vulnerable natural resources are not protected against the triple crises, the further process for turning those resources into meals and nutrition supplies is cut off. Thus, natural resources are identified as vulnerable areas under crises; the social protection priorities and measures of the natural resources need to be identified based on local geographic and agricultural features.
5) *Agriculturalabandonment inaffectedzone*: “Agricultural abandonment” threatens food security by limiting the production ability of the local food system. During crises, farmers could intentionally or unintentionally cease farming activities and give away agricultural land for natural encroachment [10,54,[59], [60], [61], [62]]. This could further result in the discontinued supply of agricultural products and lead to limited local processed food and nutrition supply [29,33,34,45,46,52,63,64]. Without external supplies, the local community would inevitably be trapped in the vicious cycle of food insecurity. Thus, the community of productive farmers is identified as the food system's key area of vulnerability under triple crises, calling for protection and gender equitable programs and measures.
6) *Unregulatedfoodprocessing*: “Unregulated food processing” harms food security with inconsistent quality of food. Under regular settings without crises, food regulation is implemented for lowering the social burden caused by food poisoning or other food-borne diseases [17,26,[65], [66], [67], [68], [69]]. As regulation weakens under crises, local communities often lower the standard for food, meal, and nutrition supply to avoid the feeling of hunger. Compared to healthy adults, the population with high nutrition demands is more susceptible to malnutrition and has severe health consequences resulting from food poison or other food-borne diseases [[70], [71], [72], [73], [74]]. Thus, we identify infants, pregnant women, people living with non-communicable diseases and the elderly as vulnerable groups under triple crises, urging nutrition support for them in conquering the vicious cycle of food insecurity and poverty.
7) *Poverty:* Communities trapped in “poverty” without social support has limited access to quality food and nutrition supply, and could “fall out of the food system”. Under crises, the majority of the population are focused on living an unfamiliar life without a regular paycheck [34,38,59,61,[75], [76], [77], [78], [79]]. Without proper support and guidance, “poverty” not only leads to malnutrition but also fuels violent behaviors due to confusion and uncertainty [18,34,54]. Thus, we identify the unemployed productive adults also as a vulnerable segment within food systems under triple crises, needed targeted social protection policies and programs, such as psychosocial support, retraining and upskilling collective resilience and equity programs for affected communities and globally.

The analytical model presented here, aims to build synergy between actions from different stakeholders by identifying the most vulnerable part of the food system under crises. Translating shared commitment into more resilient and green actions and deliverables aligned with the vision of “The Africa we want” relies on systemic cooperation and connections of food security and agriculture production, while addressing local farmers’ livelihoods consequences [80]. This cooperation can be enabled through mechanisms such as the Paris Agreement proposed annual 100 billion USD in climate financing from developed countries to enable green energy transitions and climate adaptation investments in developing countries [81]. In the following paragraph, we further examine the existing solutions to strengthen vulnerable areas and build food system resilience leadership and financing mechanisms through synergistic commitment of global and local stakeholders, and to create more stable, resilient, equitable and green food systems transformation.

### 2.3 Innovative approaches to food system strengthening against the triple crises in Africa

Our results showed that there is an urgent need to address the food insecurity through collective, robust, and sustainable alliance amongst diverse stakeholders and sectors. This involves implementing more productive, equitable climate change adaptation and mitigation tactics, which will drive lasting innovation in climate-sensitive agriculture and community-targeted poverty alleviation packages. Ultimately, innovative approaches are needed to strengthen the food system for future generations through climate sensitive and green innovations, entrepreneurship, leadership, capacity development, and increased connection of farmers, agribusinesses and urban markers to create thriving value chains, frost equitable livelihoods and promote prosperity.

To build food system resilience, innovative and systematic approaches are needed to mitigate the seven identified “pathways of negative impacts” and slow or even stop the vicious cycle of food insecurity. Our results revealed that Africa's food system(s) remains significantly vulnerable to the effects of climate change, conflicts, and pandemics with scattered response efforts proposed by different stakeholders. It is extremely critical to take a One Health approach for strengthening the food system with coordination, collaboration, capacity building, and communication at all levels of human-animal-environment sectors.

Coordinated investment in building resilience is crucial for sustainably improving food and nutrition security governance across Africa. This requires increased focus on prevention and preparedness for food crises, along with contextual strategies and finance projects that strengthen local food systems. However, Africa has only met 3% of the required green finance investment for promoting socioeconomic growth, and reducing poverty across the continent. Harnessing innovative spirit and research needs requires urgent innovative financing to power the continent's sustainable, resilient, and inclusive food systems adapted to local contexts. This plays a pivotal role in refining and disseminating collaborative strategies for enhancing the productivity, sustainability, and resilience of food systems in Africa. Climate-resilient agricultural practices are essential through multi-sector partnerships and collaborative efforts; especially, blending local knowledge with modern technologies which can optimize productivity, advocate for policy changes, promote knowledge sharing. Coordinated support for sustainable and resilient food systems, achieved through the promotion of sustainable investments and climate-resistant practices, is needed to enable policy reforms and transparent resource allocation in sustainability, inclusivity, and prosperity.

Promoting concerted One Health food security and nutrition leadership commitment and efforts for peace, conflict resolution and stable development in Africa is needed. This can be achieved through strengthening local and national to regional agricultural and food systems research and innovations, fostering more cooperative and productive human development through capacity building, and implementing equitable food and social protection programs. Moreover, it is essential to prioritize climate sensitive and resilient community-based projects, establish strong multisectoral collaboration, embrace inclusiveness and social cohesion approaches, and implement interventions to withstand food insecurity threats. Bold and programmatic actions are required to ensure food security for the achievement of Africa 2063 and Sustainable Development Goals 2030 agenda.

Harnessing acccess to and uptake of new technologies and tools has been promoted to be the key gamechanger for strengthening food system and transforming agro-businesses globally. Among these emerging technologies, Artificial intelligence (AI) and Deep Learning Models (DLMs) stand out as having great potential to transform food systems and provide sustainable solutions for food security. The innovation is continuing to be regard as the renewable power to sustainable growth. Assessing how nascent technologies like AI, DLMs, blockchain, drones and robotics can provide operational solutions in the constantly changing environment, as under climate change, conflict, and COVID-19 crises, and optimizing interventions across various situations. Importantly, by aggregating data to determine what to collect, and how to distribute it in a timely and cost-effective way, we can significantly increase agriculture production and productivity, thereby addressing food insecurity and poverty/hunger alleviation. Specifically, AI has rapidly evolved, becoming increasingly capable of solving complex food systems problems, including issues related to health and food and nutrition insecurity. With AI, data and data-driven decisions has become the new game-changer and key differentiator, empowering companies and firms to enhance their operations and product development with a higher chance of success, leading to socioeconomic development. This data-driven transformation in both society and corporate practices must be driven by agricultural biodiversity and led by empowered local food producers and consumers, fostering behavioral change and resilience to achieve the UN Sustainable Development Goals of “Zero Hunger”, which emphasizes the need to build resilience for food security and global nutrition. These technologies can also help facilitate models for better, informed, and quick decision-making in the complex, ever-changing environment of food insecurity, ending hunger, improving nutrition, and promoting sustainable agriculture, and generating multi-pronged process financial predictions.

Transparency and effective communication among different stakeholders are essential in building trust and for prioritizing adaptations to climate risks adaptations and resilient agricultural production at scale. Building natural resource development platform and framework can be strategic for enhance food and nutrition resilience and equitable strategies for all against undue competition and scarcity. National and regional food system strengthening process can enable the expanding of market access, economic growth and improving famers’ productivity; therefore, it is essential to strengthen community-based One Health food systems programs and interventions by providing technical assistance, leveraging existing and new food system partnerships and networks.

Here, the vulnerabilities of food insecurity identified in Table 2, as a conceptual framework, reveal the actionable areas in the complex system of triple-crises-induced food insecurity. Based on literature reviewed, structured suggestions for food system resilience building in Africa have been proposed under its framework, illustrating how One Health approach can be optimized to guide food system strengthening under crises, to improve access to and use of food and agricultural commodities support, to ensure food security, to alleviate nutrition-related poverty, and to strengthen supply chain management.

**Table 2:** The resilience building approach against the triple-crises of food security in Africa.

Areas of vulnerability	Related One Health sector	Recommendations
Evidence-informed transformative change and governance	Environment & Human & Animal	Adopt One Health thinking, reforms, and investment for identifying shortage within food supply chains. Invest in climate-sensitive innovations and Increasing access to resources and financial system
Food-related showcases and lessons learned including supply chain management	Animal & Human	Strengthen and guard food transportation both nationally and internationally.
Local food production entities and inclusivity	Animal & Human	Youths' voices and opportunities Extra protection to those local entities despite size and ownership. Ecofriendly appliance and technology transfer
Natural resources	Animal & Environment	Specific natural resources requiring attention need to be identified based on local geographic and agricultural features.
Productive and resilient farmers partnership	Environment & Human & Animal	Provide support and protection to local farmers.
Population with high nutrition demands and gender-empowerment	Human	Provide external nutrition support to infants, pregnant women, people living with non-communicable diseases, and the elderly
Unemployed productive adults	Human	Provide proper social support.

## 3 Discussion

The results of this review highlight the potential of One Health approach in understanding and solving crises-induced food insecurity in Africa. This approach recognizes that food security is not solely a human issue but is also influenced by factors such as animal health, ecosystem health, and the overall sustainability of food systems. By considering these interconnected factors, we provided conceptual models that capture the complex nature of the issue and aim to facilitate consensus and synergy for strengthening the food system under triple crises.

Building consensus on food system strengthening under crises can be a challenging task, as it requires bringing together various stakeholders with diverse perspectives and interests [82,83]. The conceptual model, generated with our review, provides a visual representation of the interconnected pathways between the triple crises and food insecurity. It helps stakeholders understand the complex dynamics at play and how different elements within the food system are impacted. This shared understanding creates a foundation for consensus building by establishing a common language and framework for discussions. Furthermore, identifying the seven vulnerability areas within the food systems allows stakeholders to prioritize and focus their efforts on the most critical aspects affected by the crises. This targeted approach helps in narrowing down the scope of discussions and actions, making it easier to find common ground and build consensus around specific areas that require immediate attention.

In response to the identified vulnerabilities under limited resources, the synergizing of innovative approaches is crucial for strengthening the food system against the triple crises in Africa [82,84]. The results of this review emphasize the urgency of adopting One Health approach, which entails coordination, collaboration, capacity building, and communication across the human-animal-environment sectors. By integrating these different sectors, the One Health approach can guide efforts to enhance food system resilience, improve access to agricultural commodities, ensure food security, reduce nutrition-related poverty, and strengthen supply chain management.

Consensus and synergy among stakeholders are essential under crises [83,84]. The One Health approach, with its focus on the interconnectedness of human, animal, and environmental health, provides a framework for food systems integration and collaboration. Through consensus building, vulnerability prioritization, and targeted actions, stakeholders can work together to build a more resilient and sustainable food system in Africa. This collaborative effort can further ensure food security and mitigate the impacts of triple crises on the food system.

## 4 Recommendations for an enabling environment

Despite the potential of the One Health approach, several challenges need to be addressed for its successful implementation. To create an environment that supports the One Health approach and effectively addresses the food insecurity and malnutrition/hunger challenges in Africa caused by multiple crises, additional recommendations and actions aiming at enhancing smart policies, collaboration, and strategic actions among stakeholders at the international and national levels are highlighted:

1) *Develop innovative partnerships and investment strategies to combat the impacts of climate change on food systems and food security*.
  • Maximizing the potential of African Continental Free Trade Area agreement to boost economic growth, diversify economies, and strengthen food safety systems, while encouraging the diversify of funding sources by welcoming other public-private investment opportunities.
  • Investing in climate-smart agriculture strategies and food systems research collaboration, to further address the growing African population's pressure on global resources and food welfare by implementing innovative farming solutions and capacity building to empower global food sustainability and achieve the goal of ‘no child goes to bed hungry’ by 2030.
  • Providing more opportunities, including employment and capacity development, to support local communities and strengthen the food system; while prioritizing the consolidation of democracy, good governance, and resilient health systems to ensure food security and achieve universal health coverage.
2) *Invest in local-led and community-driven initiatives for building local and regional robust and resilient food systems*.
  • Turning off food insecurity-related crises effects through local transformation and recovery business approaches.
  • Engaging communities in the production and distribution of quality food through regenerative crops and sustainable food sources and supply chain management.
  • Supporting agricfood innovative solutions such as co-investment and co-production models, 3D food, farming mechanization and personalized nutrition.
  • Focused industrialization and exportation: Developing targeted industrial sectors related to food production can enhance productivity and strengthen the food trading system.
  • Green and nature-based solutions: Implementing environmental-friendly approaches in agriculture and food production and waste management optimization can contribute to sustainable and resilient food systems.
  • Digital technologies adoption and implementation: Embracing digital solutions fidelity and sustainability can improve efficiency, traceability, and access to information in the food system resiliency, markets and economic growth.
3) *Enhancing One Health governance and capacities, to address the impacts of crises like the COVID-19 pandemic and conflicts on national economies and food security through innovative approaches and evidence-based decisions*.
  • Increasing investment in research and development of One Health strategies to mitigate risks associated with climate change, droughts, floods, and new pests. Advocating for the importance of ecology and human-centered regulations to address Africa's developmental challenges and enhance local agriculture production.
  • Prioritizing comprehensive One Health surveillance, response programs, and governance to address emerging infectious diseases and reduce risks.
  • Strengthening open access food and nutrition safety data and databases to increase public trust, promote sustainable food systems, and support evidence-based policies.

## 5 Conclusion

Climate change, COVID-19 pandemic and conflict crises continue to adversely affect climate-induced food systems shocks and to aggravate health-related emergencies crisis in Africa and elsewhere. Despite the need for more secure and sustainable food systems significant evidence gaps and challenges exist and persist due to nexus of the triple emergencies on production, supply chains and productivity. Adopting evidence-based policies, resilient and sustainable food security and food systems approaches is essential in shaping and transforming food systems for achieving the SDGs, 2030 and Africa Union Agenda 2063. Innovative long-term commitment and partnerships with traditional and international stakeholders can yield the desired outcomes. Building national and community-based contextual, resilient, and transformative food systems and farming policies and programs is paramount to strengthening climate adaptation, social cohesion and inclusiveness and social protection capacities towards overall livelihoods.

Effective and timely One Health trans-disciplinary strategies and measures are urgently needed to prevent and control zoonotic diseases outbreaks, conflicts and pandemic threats, issues of hunger and poverty to shared benefits and prosperous returns. Recognizing the impact and capacity to prepare and respond to global/local health security underscored the current fund allocation commitment and collective assistance funding in the continent. Africa region continues to be challenged by unprecedented outbreaks and shocks crises. Strengthening capacity for effective participation in policy dialogues and mitigation networks on the impact of ongoing and future complex crises.

Recommendations highlight prospective solutions including implementing local evidence-based food production systems and food security strategies, increasing investment in climate smart agriculture and food systems innovations through promoting climate adaptation and clean energy solutions, accelerating climate long-term food security policies and resilience solutions. Moreover, investing and boosting public-private partnership and research collaboration implementing COVID-19 food and agriculture resilience and recovery programs. Importantly, building more robust and resilient partnerships in advocacy and scaling up innovative-based approaches to agriculture and food systems technologies and green climate change ecosystem solutions, while promoting evidence-based healthier and sustainable food choices and food supply chain resilience, as well as community-based food systems resilience projects across Africa.

## Funding

The researchers are grateful to Africa Disease Intelligence, Preparedness and Response for the enabling research environment and administrative support.

## Declaration of competing interest

The authors declare that they have no known competing financial interests or personal relationships that could have appeared to influence the work reported in this paper.
