# Supplementary material for: Triple-crises-induced food insecurity: systematic understanding and resilience building approaches in Africa
Source: Sci One Health. 2023 Oct 29;2:100044. doi: 10.1016/j.soh.2023.100044 (PMC11262256; doi:10.1016/j.soh.2023.100044)
Supplement: Multimedia component 1 [file mmc1.docx]

Annex 1 Protocol of the One-Health-thinking-guided Literature Review

In the following paragraphs, we present how this review, guided by One Health thinking, select a theoretical framework (of the *Theory of Change*) to understand the complex process leading to the crises-induced food insecurity in the African region, and also identify the key areas for actions based on the *Vulnerability/Resilience Theory*.

One Health Thinking and Theory of Change

Guided by the One Health thinking, crises-induced food insecurity in Africa is regarded as, a complex interaction of human, animal, plant and environment leading to the disruption of food system(s) in African region. Defined by the OHHLEP (One Health High-Level Expert Panel) of the WHO (World Health Organization), One Health is “grounded on the understanding that human health is closely linked to the healthiness of food, animals and the environment, and the healthy balance of their impact on the ecosystems they share, everywhere in the world” [1]. To address the crises-induced food induced food insecurity, One Health thinking guides the study to first identify the complex nature of crises-induced food insecurity with help from the Theory of Change.

Theory of Change is adopted to logically demonstrate how triple crises might cause food insecurity. Theory of Change (ToC) is a useful tool for understanding complex systems (such as food systems), as well as external impacts (such as triple crises) and their caused changes (such as crises-induced food insecurity). Originally developed as a tool for clarifying the assumptions and relationships underlying social interventions in the field of program evaluation, it provides a logical framework for our studies to reveal how the various components of the crises might produce the food insecurity. [1–3]

With adoption of the Theory of Change, the complex relationship between food insecurity and triple crises has the following premises. Food insecurity, the disruption of the food system(s), is regarded as the outcome of crises; and hunger and malnutrition in the population as the output of the crises. The process causing food insecurity can therefore be conceptualized as a dynamic process, in which crises can make impacts through pathways of change which leads to food insecurity, and furthermore the hunger and malnutrition in population. In this research, we’ll refer to this process as the “pathways of impacts” [2].

For visualization of the process of triple-crises-induced food insecurity, the Logic Model is adopted. As an illustration tool of the Theory of Change, Logic Model is to “graphically illustrate the components (inputs, activities, outputs, outcomes, impacts)” of the “pathways of impacts” in a structured, and logical way [2].

Literature-Review-Generated Logic Model

Grounded by the Theory of Change, the aim of the literature review is not only to demonstrate the outputs, outcomes, and impact of the triple crises, but also to outline the linkages. Each effect of the triple crises is therefore shown in a logical relationship to all the others by the Logical Model of the triple-crises-induced food insecurity [2].

To meet this end, we searched peer-reviewed articles within PubMed with the following keywords:

*("Crises" or "Climate Change" or "Conflict" or "Pandemics") and ("food security" or "food system" or "food supply chain" or "nutrition") and ("Africa*" or "Sahara*" or "Eritrea" or "Eswatini" or "Swaziland" or "Ethiopia" or "Gabon" or "Gambia" or "Ghana" or "Guinea" or "Guinea-Bissau" or "Kenya" or "Lesotho" or "Liberia" or "Libya" or "Madagascar" or "Malawi" or "Mali" or "Mauritania" or "Mauritius" or "Morocco" or "Mozambique" or "Namibia" or "Niger" or "Nigeria" or "Rwanda" or "São Tomé*" or "Senegal" or "Seychelles" or "Sierra Leone" or "Somalia" or "South Africa" or "South Sudan" or "Sudan" or "Tanzania" or "Togo" or "Tunisia" or "Uganda" or "Zambia" or "Zimbabwe").*

The literature included is with descriptions and empirical evidence on how the crises in concern (Climate change, Conflicts, and Pandemics such as COVID-19) negatively impact the food system(s) in Africa. The search period is limited to within recent 3 years (2019-2022), to ensure the timeliness of the literature. In total 3,426 articles were returned from the search; all article’s titles were scanned for relavency and duplication, and yielded 302 articles; with closer examination, 43 articles concerns the long-term impact of crises-induced food insecurity, 92/17/56 articles concerns the impact of climate change/conflict/pandemic on food system, and 103 articles offering specific solutions on crises-induced food insecurity. The selection of the literature is documented in *Annex 2*; all 302 of included literature are reviewed and managed via Zotero (accessible via https://www.zotero.org/groups/reviewed_for_crises_induced_food_insecurity).

For each literature present the impact of climate change/conflict/pandemic on food system, semiotic analysis is conducted to identify the components of the “pathways of impacts” and the directional relationship among components. Semiotic Analysis is conducted and documented in Zotero. With the “comment” feature of Zotero, the analyst marked components of the pathways within literature, and organized them into relational components.

As the returns of the literature search via PubMed are listed by relevancy to the search terms, the literature collection process and semiotic analysis were conducted iteratively (one literature at a time), until the semiotic analysis displayed information in repetition.

Build the Actionable Roadmap for Resilience Building with Vulnerability/Resilience Theory

The framework of Vulnerability is adopted to identify the key actionable areas to tackle the triple-crises-induced food insecurity in the African region. In the field of Disaster Management, vulnerability is referred to as the part of the social system which is most susceptible to crises; and the social adaptation to the crises is determined by the proper protection of the most vulnerable parts of the system. Hence, the identification of the vulnerabilities under crises are the key to system resilience building [4,5].

For our research, the vulnerabilities of the food system under the triple crises are identified with help from the Logic Model. According to the Vulnerability Theory, the vulnerabilities of the food system under crises can be conceptualized as the possible “impact points” of crises on the food system. Those “impact points” are the end of each “pathway of impact”, graphically presented in the Logic Model of the triple-crises-induced food insecurity.

With the Theory of Change visualizing the complex nature of triple-crises-induced food insecurity, and the Vulnerability Theory directing the actionable points to prevent triple-crises-induced food insecurity, we can “find the needles in a haystack” -- distinguishing the parts of the food system that urgently need protection, thus forming an actionable roadmap for tackling vulnerabilities and building resilience of the food system against the triple crises.

Limitations on Literature Review Strategy

The literature review though stickily followed the protocol is still limited by its lack of subjectivity and repeatability. The Logical Model is formed according to semiotic analysis, which cannot subjectively present a fix reality on the criese-induced changes of food systems; as the understandings on the “impact” are varied among existing literature, the generalization of the “impact pathways” is guided by our pre-understanding of *Theory of Change* stated above. To increase the subjectivity of the results, the final version of the logical model has been reviewed by several subject matter experts for validation. Within vast majority of literature in food security, the terms selected for the logic model have various synonyms creating barriers for repeatability of our review attempt. To have better representation, the terms used in the final manuscript have been assimilated in nouns or gerunds; all literature reviewed are presented in Annex 2 and the open Zotero group (accessible via https://www.zotero.org/groups/reviewed_for_crises_induced_food_insecurity).

**Reference**

[1]One Health High-Level Expert Panel (OHHLEP), Adisasmito WB, Almuhairi S, Behravesh CB, Bilivogui P, Bukachi SA, et al. One Health: A new definition for a sustainable and healthy future. PLOS Pathog 2022;18:e1010537. https://doi.org/10.1371/journal.ppat.1010537.

[2]Rüegg SR, Nielsen LR, Buttigieg SC, Santa M, Aragrande M, Canali M, et al. A Systems Approach to Evaluate One Health Initiatives. Front Vet Sci 2018;5:23. https://doi.org/10.3389/fvets.2018.00023.

[3]Connell JP, Kubisch AC. Applying a Theory of Change Approach to the Evaluation of Comprehensive Community Initiatives: Progress, Prospects, and Problems n.d.

[4]Folke C. Resilience: The emergence of a perspective for social–ecological systems analyses. Glob Environ Change 2006;16:253–67. https://doi.org/10.1016/j.gloenvcha.2006.04.002.

[5]Miller F, Osbahr H, Boyd E, Thomalla F, Bharwani S, Ziervogel G, et al. Resilience and Vulnerability: Complementary or Conflicting Concepts? Ecol Soc 2010;15:art11. https://doi.org/10.5751/ES-03378-150311.
